# The cooperation between orf virus and *Staphylococcus aureus* leads to intractable lesions in skin infection

**DOI:** 10.3389/fcimb.2023.1213694

**Published:** 2024-01-08

**Authors:** Yongzhong Yu, Yudong Cui, Baifen Song

**Affiliations:** ^1^ College of Biological Science and Technology, Heilongjiang Bayi Agricultural University, Daqing, China; ^2^ College of Veterinary Medicine, China Agricultural University, Beijing, China

**Keywords:** Orf virus, *Staphylococcus aureus*, secondary infections, damage superposition, pathologic mechanism

## Abstract

A large amount of evidence shows that different kinds of microorganisms can jointly cope with environmental pressures including cell hosts. For example, in many cases, it has been found that secondary or mixed infection of animals caused by ORFV (an epitheliophilic Parapoxvirus) and bacteria (such as Staphylococcus aureus or Streptococcus) shows a mutual aid mode that indirectly leads to the deterioration of the disease. However, the lack of research on the co-pathogenic mechanism, including how to hijack and destroy the cell host in the pathological microenvironment, has hindered the in-depth understanding of the pathogenic process and consequences of this complex infection and the development of clinical treatment methods. Here, we summarized the current strategies of trapping cell hosts together, based on the previously defined ORFV-Host (O-H) system. The opportunistic invasion of *S. aureus* destroyed the delicate dynamic balance of the O-H, thus aggravating tissue damage through bacterial products (mediated by Agr), even causing sepsis or inducing cytokine storms. In fact, the virus products from its adaptive regulatory system (VARS) weaken the immune attacks and block molecular pathways, so that *S. aureus* can settle there more smoothly, and the toxins can penetrate into local tissues more quickly. This paper focuses on the main challenges faced by cell hosts in dealing with mixed infection, which provides a starting point for us to deal with this disease in the future.

## The establishment of O-H system can easily cause secondary infection

Orf is affecting mostly small ruminants including sheep and goats and, sometimes, wild animals (Hosamani et al., 2009). ORFV is a representative member of the genus *Parapoxvirus* (PPV) of the *Poxviridae* family in virus taxonomy (Matthews, 1982), with zoonotic importance to humans. PPVs can occasionally cross-infect other species, such as camels and deer. ORFV infection gives rise to contagious ecthyma in damaged skin (Mercer et al., 2006), but rarely causes systemic transmission (Robinson and Lyttle, 1992) unless exerting secondary infection or mixed infection.

**Figure 1:**
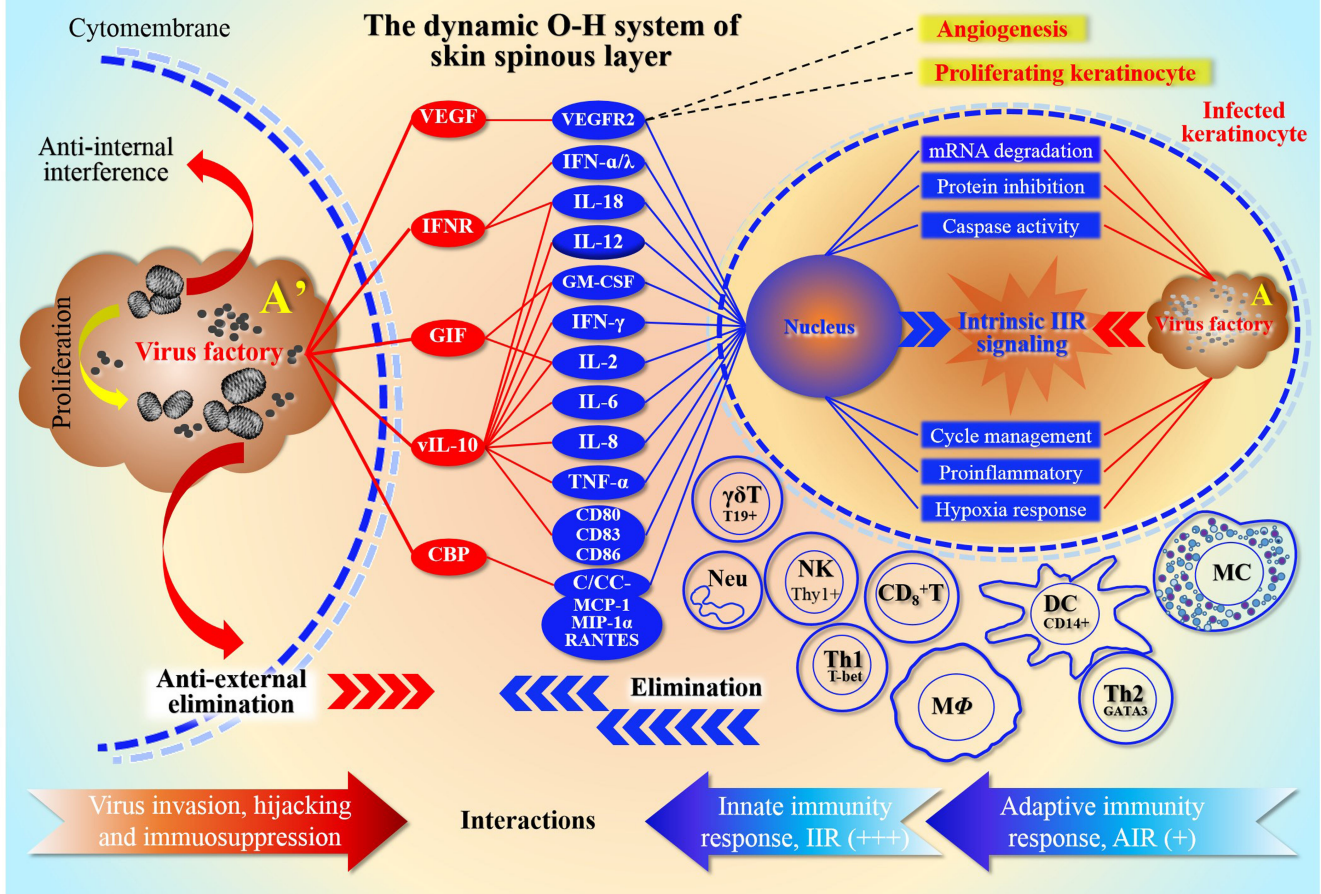
Schematic representation of the O-H system depth resolution The relationship between ORFV host antagonism and competition in the O-H system is described in detail. The diagram shows the form of the array of camp members represented by both sides. It reflects the ability of the two parties to mobilize their own “effectors”, and also reflects the dependence of both parties on these stress effectors. For ORFV, in order to protect the virus factory from immune elimination caused by infection, they attempt to interfere with multiple stress levels of host, mainly by modulating IIR and AIR. The top left side of the figure represents the antiviral effect of ORFV in response to extracellular cells. Red oval color blocks represent viral components, except that VEGF separately antagonize the effector molecules secreted by the host cells, thereby inhibiting the immune cell activation, and subsequently weakening the levels of native and adaptive immunity. The main function of VEGF is to bind its cellular receptor VEGFR2, which in turn promotes cell proliferation and neovascularization. The top right of the figure represents the focus of competition between virus and host intracellular. In fact, itrsquo;s a duel between the genetic information encoded by the nucleic acids within the nucleus and the genetic information in the viral factory. The various activated immune cells in the middle and lower parts of the right panel are the cells mainly involved in defense, and they are induced by the virus-infected KC, including natural immune cells and lymphocytes. The red and blue arrows at the bottom of the figure, represent the neutralization relationships of viral and host immune responses, respectively. Among them, the natural immunity is more active (signed as +++), while in the specific immunity stage, only Th1 cells are more active (signed as +); because the antigen-presenting cells (such as macrophages and dendritic cells) are inhibited by the virus, then the antibody secretion and function are greatly affected. The number of plus signs represents the strength of the effect magnitude. Here note that Arsquo; is an enlarged plot of A, representing the enlarged virus factory map on the right in the infected KC.

Previously, we defined an ORFV-Host (O-H) system, namely O-H, and explained the potential dynamic relationship and mutual adaptation between them (Yu et al., 2022). During infection, ORFV attempts to protect the virus factory from immune elimination by interfering with host cells, mainly through reversely modulating innate immunity response (IIR) and adaptive immunity response (AIR) (**Figure 1**). Virus factories are key elements of the O-H in plan for adaptation to host environments. Therefore, this system seems to be a device reflecting the relationship of the elimination and anti-elimination. With the survival strategy, O-H is carefully managed by viruses, and directs a large number of interactions when experiencing IIR and AIR. For this purpose, the virus mainly interferes with the effective operation of the signal pathways, such as 2’-5’oligoadenylate synthetase(OAS)/RNase L, intracellularly (Yu et al., 2022). However, extracellular cytokines are the main targets of virus evasion mechanisms (Hernaez and Alcam, 2020). Therefore, poxviruses express several decoy-receptors of their cellular counterparts, such as vIL-18BP, vIFN-IBP and CrmD, *etc.* to sequester or neutralize cytokines IL-1-β, TNF or/and IFN-I (Hernaez and Alcam, 2020). As mentioned above, to propagate and spread, viruses must counteract host innate immunity (Silverman, 2015). For returning to homeostasis, host cells must activate the emergency mechanism and mobilize local cells to inhibit the infection. A number of viral genes were expressed and acted within infected cells to reversely inhibit the anti-viral host response from IIR and AIR effects. To facilitate evasion, viruses try to inactivate the stress response of host cells as needed.

In addition to constitutive structural genes, the viral immunomodulators are responsible for weakening immune stimulation and maintaining the development of infection on the premise of maintaining O-H homeostasis. Of course, the regulatory ability of virus to overcome host pressure goes far beyond these aspects (Yu et al., 2022). The immunomodulators support the distinct genus-species characteristics of the virus which are different from those of the *orthopoxvirus*. Generally, these genes are selectively captured by viruses in order to adapt to the host environment (Fleming et al., 2015), and are armed after deliberate adaptation to host. Today, we can modify the composition of these genes artificially, so as to introduce foreign genes and develop several dynamic gene delivery facilities (potential alternative vectors), such as design of virus-based therapeutic vaccine (Rziha et al., 2000; Amann et al., 2013; Schneider et al., 2020). Unfortunately, the homeostasis of O-H can be easily disturbed by subsequent opportunistic invaders such as *S. aureus* or *Streptococcus*. The situation of secondary infection will make the condition extremely complicated, and even lead to systemic lesions.

## *S. aureus* can destroy O-H and worsen the host microenvironment in secondary infection of orf

In recent decades, the pathogenicity of bacterial secondary infection in human and animal patients has increased rapidly (Pastor et al., 2020). This has aroused people’s concerns about the health and well-being of herds, environment and biological safety. Orf is such a disease that affects the skin, creating a huge gap for *S. aureus* and also disrupting its O-H program (Chi et al., 2017). This is emphasized by the high detection rate of *S. aureus* in secondary infection (Pastor et al., 2020). Accordingly, orf can create great convenience for the secondary infection of *S. aureus*.

So far, there have been many reports and references about the secondary infection of orf, but there are few systematic studies. Recently, some scholars have made a detailed analysis of the species and quantities of bacteria secondary to orf infection (Pastor et al., 2020). The statistical data had proved that the incidence of secondary infection of orf was 42%, and the detection rate of *S. aureus* was as high as 58% in the cases of secondary infection. In addition, 30% of secondary infections can detect more than two kinds of bacteria (Pastor et al., 2020). Although the biological materials were only collected from five flocks of sheep in Romania, they were very representative of the worldwide prevalence.

**Figure 2:**
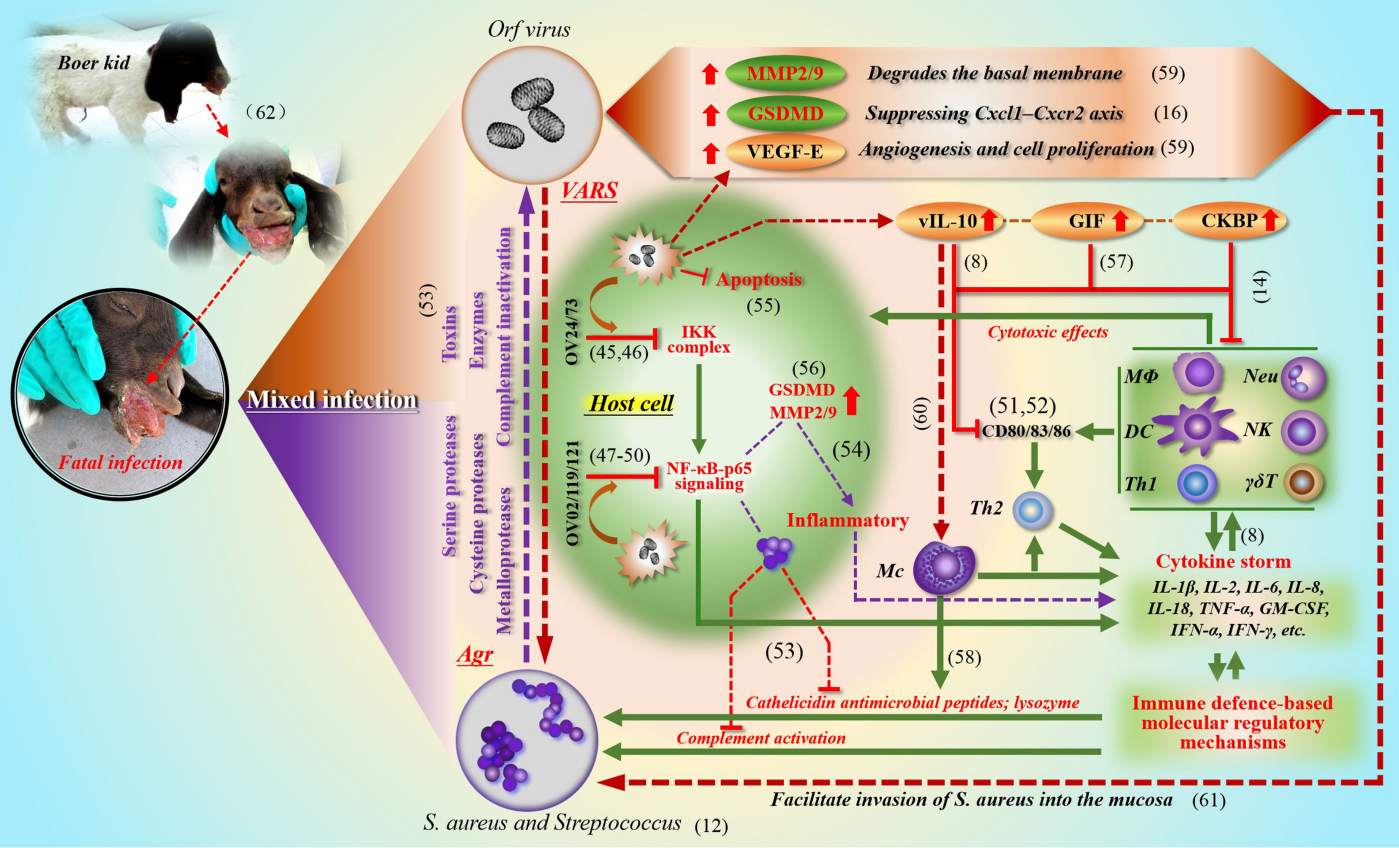
The synergistic effect of ORFV (VARS) and *S. aureus* (Agr) in a mixed infection This picture describes in detail the cooperation between ORFV (VARS) and *S. aureus (Agr)* in and out of the cell during co-infection to cope with the immune clearance effect of the host (the green part). At present, the relevant evidence of mutual benefit can be discovered in the clinical cases of co-infection of ORFV with *Staphylococcus* and *Streptococcus*. ORFV limits the activity of various cells (through IKK and NF-κB signaling; various virulence factors and immune regulatory molecules) (the red brown part), which facilitates the expansion and penetration of bacteria; and bacteria can also promote the local spread of viruses (the purple part). (Chan et al., 2006; Chen et al., 2016; Couñago et al, 2015; Diel et al., 2010; Diel et al., 2011; Fleming et al, 2015; Haig, 2006; Imlach et al., 2002; Khatiwada et al., 2017; Lateef et al., 2003; Liu et al, 2021; Nagendraprabhu et al., 2017; Nardo et al., 2008; Ning et al., 2013; Pastor et al, 2020; Shi et al., 2015; Wang et al., 2010; Westphal et al., 2009; Wise et al., 2012; Yu et al., 2020; Zecconi and Scali, 2013; Zhao et al., 2016).

Through pathological examination, it was found that the secondary infection lesions were difficult to repair themselves, and serious ulcers and gangrene appeared at any time. Compared with simple infection, the microbial reproduction of environmental spread is unprecedented. Due to the difference in individual immune level, the death threat may be approaching (see the dying Boer goat kid in **Figure 2**). In the final analysis, all this is the result of the combination of viruses and bacteria “framing” the host. A similar virus-bacteria cooperation could be found. For example, herpes simplex virus (HSV) type 1 infection promotes invasion of *S. aureus* into the epithelial tissue (Wang et al., 2012). Here, it can be seen that *S.aureus* not only disturbs the O-H, but also promotes the formation of more complicated diseases based on self-limiting orf.

During the process of mixed infection, the quantity and activity of cytokines in local lesion increased significantly. Generally, due to the excessive secretion of cytokines, the vascular permeability of the lesion site increases (in order to recruit immune cells), which makes it easier for bacteria to enter the blood vessel, and at the same time accelerates the extravasation of fluid in the blood vessel, thus destroying the tissue. All kinds of virulence factors of *S. aureus* are frequently dealt with by the host (see **Figure 2**). If left unguarded, the destruction of the epidermal barrier may lead to protein loss and facilitate the cleavage of the corneodesmosome junctions (Gomes et al., 2021)), imbalance of body fluids and electrolytes, and increased risk of local and systemic infection. Therefore, *S. aureus* is more dangerous in destroying the microenvironment of host epithelium followed by sepsis and cytokine storm.

## The molecular mechanism of synergism between orf virus and *S. aureus*

In the process of studying the molecular mechanism of *S. aureus* cooperating with ORFV to cope with host immune clearance, including IIR and AIR, we summarized the main strategies and showed the key ways of intracellular and extracellular anti-host immune effects (see **Figure 2** for details). In fact, these effects are based on well-developed regulatory mechanisms of pathogens. One is called adaptive regulatory system (VARS) in ORFV, and another one mainly comes from the *Agr* system in *S. aureus*. The extracellular enzymes of *S. aureus* regulated by *Agr*, including serine protease, cysteine protease and metalloprotease family (Dubin, 2002), play an important role in tissue damage and can also resist host immune attack. Among them, the glutamyl endopeptidase, also known as protease V8, can also degrade immunoglobulins: IgM, IgG, IgD and IgE (Miedzobrodzki et al., 1987; Takai and Ikeda, 2011; Burchacka et al., 2013), which is beneficial to the respite of the virus and the recovery of productivity. On the other hand, *S. aureus* can also up-regulate a member of the Gasdermin protein family, GSDMD, and inhibit the CXCL1-CXCR2 axis, thus hindering the recruitment of neutrophils (Liu et al., 2021). In addition, *staphylococcal* α-toxin was introduced to facilitate virus evasion from IIR and AIR (Bin et al., 2012). In return, ORFV partially alleviates cytokine activation caused by *S. aureus*. For example, CKBP of ORFV can inhibit chemokines such as CCL2, CCL3, CCL5, CXCL1, CXCL2, XCL1, and effectively prevent immune cells from gathering at the lesion site, including neutrophils, monocytes, dendritic cells (DC), mast cells and T lymphocytes (Couñago et al., 2015; Sharif et al., 2019).

In our opinion, the co-existence of ORFV and *S. aureus* in the host epithelium is an unusual warning. We also can’t ignore the protective effects of ORFV on *S. aureus* by inhibiting apoptosis and IKK complex pathway. Another prominent role is to jointly promote secretion of IL-6 (Wang and Luo, 2018) and metalloproteinases (MMP2, MMP9), and also to inhibit chemokines. IL-6 could induce abnormal differentiation of keratinocytes, and the related molecules including keratin K1, K10, filaggrin (FLG), Loricrin (Son et al., 2014), and others. Furthermore, IL-6 causes cytoskeletal changes and epidermal barrier dysfunction. It can be inferred that the cooperation is not only beneficial to the local tissue colonization of *S. aureus*, but also beneficial to the immune evasion of ORFV.

## Other aspects worthy of discussion

Recently, by phylogenetic analysis of genome, researchers found that there were highly conserved amino acid residues in the N-terminal and C-terminal domains of ORFV interferon resistance proteins (VIR) of PPVs, which was responsible for Z-DNA and dsRNA binding. The passing positions, namely N37, Y41, P57, and W59 (for Z-DNA binding) and E116, F127, F141, and K160 (for dsRNA binding), corresponded to ORFV VIR (Karki et al., 2019). This situation reflects the host-specific adaptability of ORFV. Furthermore, pathogenic bacteria such as *S. aureus* and *Streptococcus* will inevitably be introduced into the same host. In this kind of mixed infection, the collusion between the two can be described as a perfect match, and they both do their best to serve their common goal: immune evasion. For evasion from host, in the late stage of ORFV infection, virus-induced apoptosis may be beneficial to the spread of progeny virus due to the disintegration of infected cells (Castanier and Arnoult, 2011). If *S. aureus* is involved, it is believed that the trigger of apoptosis will be more decisive, because it is also beneficial to the release of bacteria.

On the other hand, we can’t just emphasize the harm of ORFV unilaterally, and let the mixed infection plot overwhelm the available value of ORFV. Administration of inactivated ORFV results in a transient stimulation of selected IIR mechanisms, which may contribute to the immune enhancement effects *in vivo* against viral and bacterial infections (Anziliero et al., 2014). This protective effect is considered to be mediated by various mechanisms of the host (Weber et al., 2013). It was balanced by additional mechanisms of infection-induced immunosuppression, which shows the regulatory ability compared to single cytokine therapy (Friebe et al., 2011). To host, the procedure of innate immune memory, that is, trained immunity, is a fundamental property of host’s defense in the immune response to a new infection (Netea et al., 2016). Gillard et al. reported that Thy1^+^ NK cells from VACV-primed mice conferred no less protection against VACV challenge in the absence of adaptive immunocytes (Gillard et al., 2011). Similarly, in a non-permissive host dog, canine blood phagocytes and T lymphocytes can be activated by inactivated ORFV (Schütze et al., 2009). If these effects are proved to be lasting, specific, predictable and clinically significant, immune training of ORFV can release comforting potential (Song and Colonna, 2018). Therefore, inactivated ORFV should be investigated further for its potential use in various anti-infection therapies (Paulsen et al., 2013; Anziliero et al., 2014; Wang and Luo, 2018). To date, some reports shows that inactivated ORFV has antiviral activity (Weber et al., 2003; Lin et al., 2015). ORFV can prevent the recurrence of genital herpes in Guinea (Ons et al., 2014). Consistent with findings in *in vivo* animal models, Friebe et al. demonstrated that inactivated ORFV acts on human immune cells, resulting in the regulation of the release of cytokines in Th1 and Th2 (Friebe et al., 2004). This is supported by the absence of severe side effects in animals and humans after single therapeutic trials (Friebe et al., 2011).

As VACV with tumor-dissolving effect, chimeric ORFV CF189 has been proved effective cytotoxicity *in vitro* and anti-tumor effect *in vivo* at a dose as low as 10^3^ PFU. These data have encouraged clinical development of this highly effective drug for triple-negative breast cancer (Choi et al., 2018). It is worth noting that perioperative administration of oncolytic ORFV and VACV can reverse NK cell inhibition (Tai et al., 2013). In addition, ORFV can be used as a carrier to produce recombinant vaccines (Tan et al., 2012; Wassie et al., 2019; Tiwari et al., 2020).

## Conclusion and perspectives

To sum up, ORFV, as a typical representative of *Parapoxvirus*, aims to construct and maintain the stability of O-H involved in co-evolution strategy with the host, so as to achieve the purpose of species transmission. However, it is often wishful thinking. When O-H is established, some pathogens will have the opportunities to invade and make ORFV an intermediary or accomplice. Here, we describe the temporary cooperation between ORFV and *S. aureus*, and deeply describe their co-pathogenic mode and molecular mechanism in response to IIR and AIR in host. Furthermore, we suggest that secondary infection or mixed infection has become an important cause of death of orf animals, reminding researchers and the public to consider it.

The topic of mixed infection of ORFV and bacteria has a long history, but it has not been paid enough attention and in-depth study in the field. Now it seems that the frequent occurrence of this disease is closely related to the welfare of animal groups and environmental safety. Therefore, it is necessary to establish effective system of disease prevention and control. In addition to good feeding management, early detection of orf is also very necessary. Especially, careful treatment of orf is important to prevent secondary infection. In addition, the use of genetic engineering technology to develop combined vaccines is also an effective way to prevent the spread of such diseases. An extremely favorable condition is ORFV’s own immune enhancement capacity and gene delivery vehicle. Thus, ORFV can be used to develop recombinant nucleic acid vaccine or biological preparation for treating secondary infection of orf for clinical application.

## Author contributions

YY, and YC. conceived and designed the manuscript. BS analyzed the data. YY. and BS. provided resources. All authors read and approved the final manuscript.
